# Notes on the genus *Ophryosporus* (Asteraceae, Eupatorieae) in Chile

**DOI:** 10.3897/phytokeys.161.53736

**Published:** 2020-09-15

**Authors:** Felix F. Merklinger, Federico Luebert

**Affiliations:** 1 University of Bonn, Nees Institute for Biodiversity of Plants, Meckenheimer Allee 170, D–53115 Bonn, Germany University of Bonn Bonn Germany; 2 Departmento de Silvicultura y Conservación de la Naturaleza, Universidad de Chile, Av. Santa Rosa 11315, Santiago, Chile Universidad de Chile Santiago Chile

**Keywords:** Atacama Desert, Compositae, endemism, lomas vegetation, Peru, species distribution, taxonomy

## Abstract

*Ophryosporus* Meyen is reviewed for Chile and an updated species list for the country based on herbarium records and literature review is presented. A key to the Chilean species is provided and a distribution range of taxa is indicated based on herbarium records and our own collections. We include several lectotypifications as well as an epitypification of *Ophryosporus hoppii*. The presence of two species, *O. hoppii* and *O. floribundus*, formerly accepted for Chile, is questioned and their actual distribution discussed.

## Abstract (es)

Se revisa el género *Ophryosporus* para Chile y se presenta una lista actualizada de especies para el país basada en registros de herbario y una revisión de la literatura. Se proporciona una clave para las especies chilenas y se indica el rango de distribución de los taxones basado en registros de herbario. Se cuestiona la presencia de dos especies, *O. hoppii* y *O. floribundus*, anteriormente aceptadas para Chile, y se discute su distribución real.

## Introduction

During field work and specimens determination as part of the collaborative research centre 1211 (http://crc1211.uni-koeln.de) – “Earth, Evolution at the Dry Limit”, we noted inconsistencies in the literature regarding the taxonomy and nomenclature of *Ophryosporus* Meyen.

**Figure 1.**
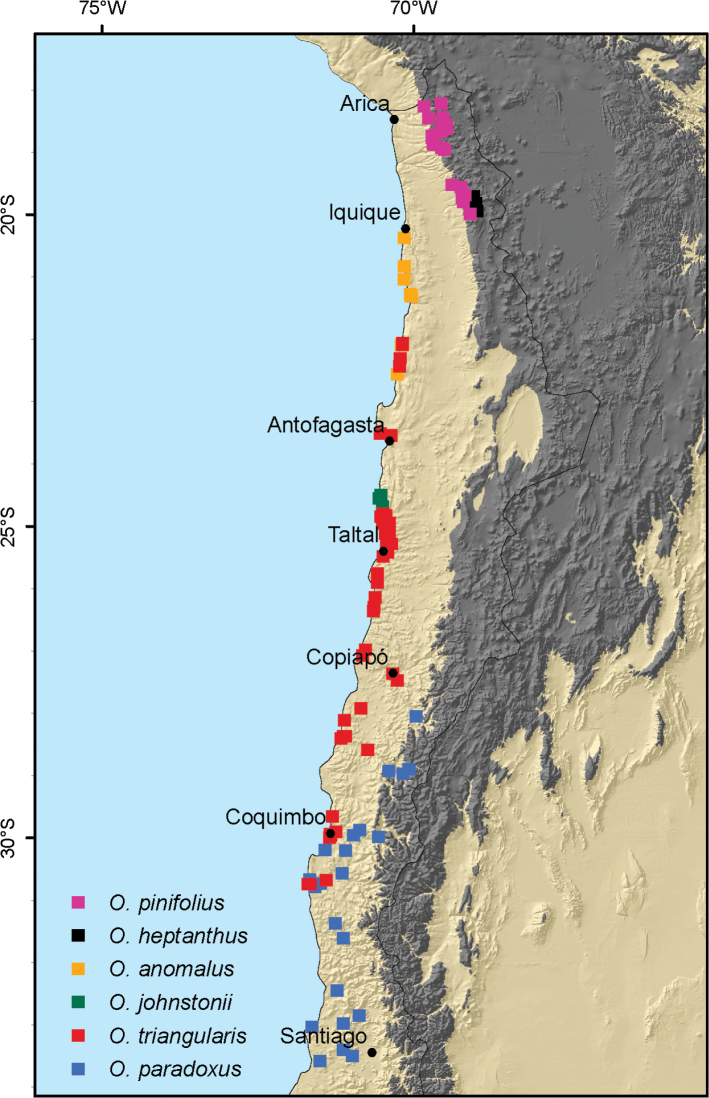
Geographical distribution of the six accepted species of *Ophryosporus* in northern Chile. Areas shaded in dark gray represent the Andean Cordillera > 3000 m elevation, areas in beige below 3000 m elevation. The black line represents the political border of Chile.

*Ophryosporus*, currently with 41 accepted species, is distributed in South America and is disjunct between the Andes from Colombia to Chile and the Atlantic Forest in southern Brazil and northern Argentina (King and Robinson 1972b). The plants are (sometimes scandent) shrubs, with usually opposite secondary branching and opposite or alternate leaves, often in fascicles. The inflorescences are corymbose or thyrsoid (Hind and Robinson 2007). The genus is further characterized by reduced anther appendages, clavate style branches, a pronounced carpopodium and distinct asymmetrical cypsela base (King and Robinson 1972a). In Chile, eight shrubby species are currently recognized (Zuloaga et al. 2008; Rodriguez et al. 2018) and they are distributed along the coast of northern Chile as well as along the Andean Cordillera, separated by the hyper arid absolute desert (Fig. 1).

An important revision of the Eupatorieae, including the genus *Ophryosporus*, was published by Robinson (1906). This work maintained two sections in the genus, section Euophryosporus including *O. triangularis* and *O. paradoxus* (Hook. & Arn.) B.D.Jacks., also a Chilean species with short internodes and thyrsoid panicles, and section Ophryochaeta B.L. Rob., including 15 species with opposite leaves, well developed internodes and capitula largely in panicles or axillary cymes. Subsequent works included some new combinations and descriptions of new species, enlarging the genus to 29 species (King and Robinson 1972a), and further additions from the genus *Piqueria* Cav. brought the total to 38 species (King and Robinson 1972b). Apart from an unpublished thesis by Plos (2012), and several lectotypifications by Plos and Sancho (2013), no recent revision for the group exists.

We were able to make extensive collections of *Ophryosporus* in Chile over a period of three years. Our survey confirmed six of the eight species reported for the country by Rodriguez et al. (2018), Luebert et al. (2007) and Zuloaga et al. (2008). We present here an updated species list of *Ophryosporus* in Chile with a detailed account of their nomenclature and distribution, and include a key for species identification.

## Methods

Field work was carried out in northern Chile and southern Peru between October 2016 and September 2019. The principal area of distribution of the Chilean species of *Ophryosporus* was covered, ranging from Valparaíso (33.05°S, type locality of *O. paradoxus*) to Arica (18.45°S) along the coast, and corresponding latitudes in the Andean cordillera of Chile. In Peru, sporadic collecting took place between Azángaro (14.92°S, type locality of *O. heptanthus* (Schultz-Bip. ex Wedd.) R.M.King & H.Rob.), Ollantaytambo (13.25°S), and in the vicinity of Lima (12°S). A total of 82 herbarium numbers were collected (Suppl. material 1: Table S1). Vouchers were deposited at the herbaria of Bonn, Germany (BONN), the Universidad de La Serena, Chile (ULS), the Universidad de Chile, Santiago (EIF) and the University of San Marcos, Lima (USM).

Specimens from the herbarium at Santiago (SGO), Leiden (L), Field Museum of Natural History (F), and Stockholm (S) were critically revised and geo-referenced to create a distribution map (Fig. 1; Suppl. material 1: Table S1). In addition, we used virtual herbaria to locate type material and online images of these were consulted where available.

**Figure 2.**
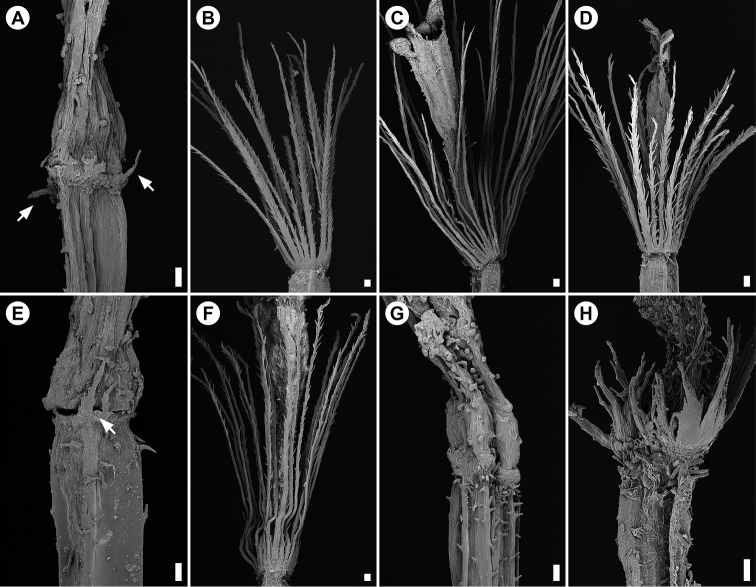
Scanning Electron Microscope (SEM) images of the apical part of the cypsela showing pappus setae/squamellae of *Ophryosporus* species. **A–F** Chilean species **G, H** Peruvian species previously referred to Chile (pappus absent in *O. floribundus*) **A***O. anomalus* (*FL 4102*) with few spreading squamellae (arrowed) **B***O. heptanthus* (*FFM 2019-20*) with conspicuous pappus setae **C***O. johnstonii* (*Johnston 5259*) with long, erect pappus setae **D***O. paradoxus* (*Zöllner 6827*) with long, erect pappus setae **E***O. pinifolius* (*FL 3452*) with small, erect squamellae (arrowed) **F***O. triangularis* (*FFM 2018-63*) with long, erect pappus setae **G***O. floribundus* (*Asplund 11079*) with no visible pappus **H***O. hoppii* (*Dillon 3926*) with squamellate pappus setae fused at the base and rather more conspicuous than in *O. pinifolius*. Scale bars: 100 μm.

Scanning Electron Microscope (SEM) images were taken of the cypselae and pappus of all taxa in question (Fig. 2). Cypselae obtained from herbarium specimens were mounted on aluminium stubs using conductive carbon cement (Leit-C, PLANO, Wetzlar, Germany) and sputter coated with gold in a sputter-coater (SCD 040, Balzers Union, Liechtenstein) for 3 minutes. Images were taken with a Stereoscan 200 electron microscope (Cambridge, England) at 15 kV.

## Results

Our results confirm six species of *Ophryosporus* in Chile. These are *O. triangularis*, *O. paradoxus*, *O. johnstonii* B.L.Rob., *O. pinifolius* (Phil.) R.M.King & H.Rob., *O. heptanthus* and *O. anomalus* R.M.King & H.Rob.; *Ophryosporus hoppii* and *O. floribundus* do not seem to occur in Chile and are in need of further taxonomic investigation.

### A key to the Chilean species of *Ophryosporus*

**Table:** 

1	Compact shrubs, erect branching to about 1 m overall height, predominantly coastal	**2**
–	Loose shrubs, branching erect to spreading, regularly exceeding 1 m in height; Andean	**5**
2	Pappus inconspicuous, formed by spreading squamellae	***O. anomalus***
–	Pappus formed by conspicuous setae	**3**
3	Leaves linear-lanceolate	***O. paradoxus***
–	Leaves triangular	**4**
4	Leaves rarely entire, usually regularly lobed or dentate, > 3 × 5 mm. Capitula pedunculated; widely distributed along the coast of northern Chile	***O. triangularis***
–	Leaves usually entire, rarely irregularly dentate, < 3 × 5 mm; Capitula sessile; known from the area between Aguada Cardón and Miguel Díaz	***O. johnstonii***
5	Leaves linear-lanceolate, pappus formed by minute 0.1–1 mm long, irregular squamellae	***O. pinifolius***
–	Leaves triangular-lanceolate, pappus formed by conspicuous setae of 3–4 mm in length	***O. heptanthus***

#### Ophryosporus anomalus

Taxon classificationPlantaeAsteralesAsteraceae

R.M.King & H.Rob., Phytologia 25: 66. 1972

38CD0A57-2CF3-516E-AE49-4A60B0331D0B

Typonym: Piqueria cumingii B.L.Rob., Proc. Amer. Acad. Arts, 42: 11. 1906, non Ophryosporus cumingii Benth. ex Baker (1895: 188, based on *Mandon 264* from Bolivia.).

##### Type.

Chile. Region I Tarapacá and Region II Antofagasta: “Peruvia meridionalis: Cobija, Iquiqui et Arica”, *H.Cuming 953* (lectotype, selected by Plos and Sancho (2013: 338): K [K000486684, photo!]; isolectotypes E [E00322766, photo!], GH [GH00010778!], K [K000486685, photo!], P [P02673192, photo!]); remaining syntypes: *Gaudichaud* s.n. (B [probably destroyed, could not be found], F [F1012247, photo!]).

*Ophryosporus anomalus* has been cited for Peru (King and Robinson 1972b; Brako and Zarucchi 1993), likely due to the type collection label (“Peruvia meridionalis...”). However, the localities mentioned there (“...Cobija, Iquiqui et Arica”) are now situated in Chile, and all other reports originate from the coastal zone around Tocopilla (Johnston 1932; Jaffuel 1936) and therefore Luebert et al. (2007) considered it a Chilean endemic. We hereby extend the distribution of the species to include the populations from the coastal area between Río Loa and Iquique, previously referred to as *O. floribundus* (Muñoz-Schick et al. 2001; Pinto and Luebert 2009). See discussion below under the latter species.

##### Specimens examined.

Chile. Region I Tarapacá: Prov. Iquique, Alto Punta Gruesa, 20°22'S, 70°09'W, 14 Dec 1997, *R. Pinto s.n.* (SGO142948); Alto Punta Patache, 20°49'S, 70°09'W, 6 Dec 1997, *R. Pinto s.n.* (SGO142949); Alto Punta Patache, 22 Jan 2000, *R. Pinto s.n.* (SGO [photo]); Alto Punta Lobos, 21°02'S, 70°09'W, 14 Jan 1998, *R. Pinto s.n.* (SGO142950); Alto Chipana, 21°16'S, 70°03'W, 15 Oct 1997, *W. Sielfeld 7* (SGO143038); Alto Chipana, 21.304528S, 70.03204W, 990 m, 21 Oct 2016, *F. Luebert, A. Stoll & T. Böhnert 3427A* (BONN, ULS); Alto Chipana, 21.292633S, 70.042234W, 840 m, 1 Oct 2019, *F. Luebert, F.F. Merklinger & J. Ruhm 4102* (BONN, EIF, K). Region II Antofagasta: Prov. Tocopilla, Tocopilla, 27 Oct 1930, *F. Jaffuel 1026* (GH); Tocopilla, Cerro Rosario, 1 Nov 1941, *M.R. Espinosa s.n.* (SGO143254); Cobija, s.a., *C. Gaudichaud s.n*. (F1012247); Cobija, quebrada Aguada Cañas, 4 Apr 1949, *W. Biese 3088* (SGO096693).

#### Ophryosporus heptanthus

Taxon classificationPlantaeAsteralesAsteraceae

(Schultz-Bip.) R.M.King & H.Rob., Phytologia 58: 528. 1985

76F68AB3-95F5-500E-AEAE-4D5FD1DEFA9C

Basionym: Eupatorium heptanthum Schultz-Bip., Bonplandia 4: 50 and 54. 1856.Eupatorium origanoides Meyen & Walp., Nov. Actorum Acad. Caes. Leop.-Carol. Nat. Cur. 19 (Suppl. material 1: Table S1): 257. 1843, nom. illeg., non Kunth (1818: 89) [Cronquistianthus origanoides (Kunth) R.M.King & H.Rob., Phytologia 23: 411. 1972].
Ophryosporus origanoides Hieron., Bot. Jahrb. Syst. 22: 707. 1897. Replacement name for Eupatorium origanoides Meyen & Walp. Type: Peru. Dept. Tacna: In planitie circa Tacoram, Apr. 1831, *Meyen s.n.* (B [probably destroyed]; F neg. 14714!).
Ophryosporus origanoides var. microcephala [as microcephalus] Hieron., Bot. Jahrb. Syst. 22: 708. 1897. Type: Bolivia. Cochabamba: 4000 m, 26 Mar 1892, *C.E.O. Kuntze s.n.* (lectotype, selected by Plos and Sancho (2013: 338): NY [NY00230826]; GH [GH00010786, photo!]).

##### Type.

Peru. Dept. Puno: “Pérou, sur les montagnes, aux environs de la ville de d’Azángaro”, *W. Lechler 1751* (lectotype, selected by Plos and Sancho (2013: 336): P [P00742426, photo!]; isolectotypes: GOET [GOET001506, photo!], K [K000542525, photo!], P [P00742425, P00742428, photo!], W [W0018472, photo!]).

This species is confirmed for Chile and has been collected by the authors near the village of Chusmiza, at the same locality as *Zöllner 2997* (L, LP) and *Gardner & Knees 6534* (E, SGO). The pappus setae of this species are formed by conspicuous yellowish-white setae of c. 4 mm in length. At this locality, *O. heptanthus* grows in local sympatry with *O. pinifolius*. However, the pappus of the latter is formed by minute squamellae and the two taxa are thus readily distinguishable. Plos (2012) cited specimen *Zöllner 2997* twice, once for *O. heptanthus* (LP) and a second time for *O. hoppii* (CONC). It is possible, that having collected both specimens at the same locality where they occur sympatrically, Zöllner mistook them for a single species and only later at the two herbaria they were identified as belonging to two distinct taxa. We could not have access to the latter material at CONC, but we assume that it corresponds either to *O. heptanthus* or *O. pinifolius*.

##### Specimens examined.

Bolivia. La Paz: Prov. Bautista Saavedra, Chajaya, a few km from Charazani, 15°13'S, 69°01'W, 3500 m, 30 Mar 1985, *J.C. Solomon 13294* (U [U1145280]). Prov. Omasuyos, Viciniis Ochachache [Achacachi], 4000 m, Jan–Apr 1859, *G. Mandon 260* (S). Prov. Murillo, 4 km up the Río Achumani from Calacoto (La Paz), 16°30'S, 68°02'W, 3600 m, 11 Apr 1986, *J.C. Solomon 15271* (U [U1145281]); 1 km NW of Ovejuyo, 16°32'S, 68°03'W, 3700–3900 m, 2 Apr 1982, *J.C. Solomon 7453* (U [U1145279]).

Chile. Region I Tarapacá: Prov. Tamarugal, Chusmiza, 3200 m, 10 Jan 1969, *O. Zöllner 2997* (L125727); quebrada de Chusmiza, 19°41'4.9"S, 69°11'01.9"W, 3350 m, 18 Feb 2003, *M.F. Gardner & S.G. Knees 6534* (E, SGO); Chusmiza, 19°40'48.2"S, 69°10'49.5"W, 3380 m, 27 Apr 2008, *M. Muñoz & A. Moreira 4940* (SGO157269); at the entrance of the Andean village Chusmiza, 19.67880S, 69.17956W, 3392 m, 2 Oct 2019, *F.F. Merklinger, F. Luebert & J. Ruhm 2019-20* (BONN, EIF, K).

Peru. Dept. Ayacucho: Prov. Lucanas, a few km from Puente Toro Muerte, 14°42'55.1"S, 74°32'44.7"W, 3589 m, 21 Mar 2019, *M. Weigend 9841/19-32* (BONN). Dept. Cusco: Prov. Cusco, alrededore Cusco, 17 May 1958, *A.L. Cabrera & H.A. Fabris 13536* (S); Río Blanco, 1500 ft [450 m], 8–19 May 1922, *J.F. Macbride & W. Featherstone 718* (S). Dept. Puno: Prov. Puno, Checayani, NE of Azángaro, 3980 m, 28 Mar 1957, *H. Ellenberg 461* (U [U1145288]); Huerta N of Puno, 3840 m, 22 Mar 1957, *H. Ellenberg 238A* (U [U1145284]); Huerta N of Puno, 4100 m, 22 Mar 1957, *H. Ellenberg 292* (U [U1145286]); Huerta N of Puno, 3840 m, 22 Mar 1957, *H. Ellenberg 238* (U [U1145285]). Prov. Lampa, Pucará, 3900 m, 22 Aug 1957, *H. Ellenberg 2753A* (U [U1145287]). Dept. Moquegua: Prov. de Mariscal Nieto, Carumas, 3200 m, 21 Feb–6 Mar 1925, *A. Weberbauer 7333* (S); Prov. General Sánchez Cerro, Puquina, outside Puquina towards Arequipa, 16°36'39.4"S, 70°11'30.8"W, 3174 m, 29 Mar 2019, *M. Weigend 9994/19-182* (BONN).

#### Ophryosporus johnstonii

Taxon classificationPlantaeAsteralesAsteraceae

B.L.Rob., Contr. Gray Herb. 77: 4. 1926

572AD3B8-6FC2-5555-B7A4-6E3257DEE485

##### Type.

Chile. Region II, Antofagasta: Prov. Antofagasta, dept. Taltal, Aguada del Panul, *I.M. Johnston 5424* (holotype: GH [GH00010781, photo!]; isotypes: S [S-R-3810!, S 10-19704!], SGO [SGO59043!]).

This peculiar species is known only from three localities north of the town of Paposo (Johnston 1929). It is distributed very narrowly within the range of *O. triangularis* and is sympatric with this latter species. Morphologically, *O. johnstonii* is very similar to *O. triangularis*.

##### Specimens examined.

Chile. Region II Antofagasta: Prov. Antofagasta, vicinity of Miguel Díaz, directly N of quebrada Iscuña, c. 55 km N of Paposo, 24°33'S, 70°33'W, 100–300 m, 15 Dec 1987, *M.O. Dillon & S. Teillier 5292* (BONN); vicinity of Aguada de Miguel Díaz, 24°35'S, 1–4 Dec 1925, *I.M. Johnston 5310* (SGO059042); vicinity of Aguada Cardón, 24°45'S, 30 Nov 1925, *I.M. Johnston 5259* (S); rocky slopes of Aguada Cardón, 24.74173S, 70.54385W, 210 m, 15 Oct 2016, *F. Luebert, A. Stoll & T. Böhnert 3384* (BONN, ULS); Aguada Cardón, 24.741717S, 70.542687W, 210 m, 5 Oct 2017, *F. Luebert, T. Böhnert & F.F. Merklinger 3950* (BONN, ULS).

#### Ophryosporus paradoxus

Taxon classificationPlantaeAsteralesAsteraceae

(Hook. & Arn.) B.D.Jacks., Index Kew. 2 (1): 354. 1894

5B4FB92D-F4CE-5E63-919C-92E700DB8779

Basionym: Eupatorium paradoxum Hook. & Arn., Compan. Bot. Mag. 1: 240. 1835.
Nothites baccharidea DC. Prodr. 5: 187. 1836.
Stevia baccharoides (DC.) Meigen., Bot. Jahrb. Syst. 17: 283. 1893. Type. Chile. Region V Valparaíso: *C.L.G. Bertero 837* (lectotype, designated here: G-DC[G00495730, photo!]; isolectotypes: G-DC [G00495717, mounted onto the same sheet as lectotype]; L [L.3661664!]).

##### Type.

Chile. Region V Valparaíso: *T.C. Bridges 52* (lectotype, selected by Plos and Sancho (2013: 336): E [E00249901, photo!]; isolectotypes: K [K486667, photo!], W [W0018468, photo!]); remaining syntypes: Valparaíso, *H. Cuming 337*, K [K486668, photo!], E [E249900, E249902, photo!]; W [W0018467, photo!]. Valparaíso, *J.Gillies* (not seen).

This is the southernmost species of *Ophryosporus* in Chile, distributed from the region Metropolitana de Santiago northward to the region Atacama. This species is not strictly limited to the coast but also occurs further inland, for example in the Cuesta Las Chilcas or Andacollo.

*Ophryosporus paradoxus* is a very distinct species that can be differentiated from *O. triangularis* by its larger, lanceolate and rather papery leaves with strongly lobed margins, as opposed to the small, triangular, slightly fleshy leaves with revolute margins in *O. triangularis*. The secondary inflorescences are thyrsoid, emerge terminally and produce florets with white corollas and a pappus of white setae up to c. 3 mm long.

##### Specimens examined.

Chile. Region III Atacama: Prov. Copiapó, Jorquera-valley, 12 Jan 1970, *O. Zöllner 4682* (L3661656). Prov. Huasco, below El Chivato, 28°54'S, 70°04'W, 1800 m, 4 Jan 1926, *I.M. Johnston 5870* (S); Resguardo, 28°58'S, 70°10'W, 1530 m, 4 Jan 1926, *I.M. Johnston 5863* (S); valley San Félix, 1180 m, 16 Dec 1941, *E. Pisano V. & R. Bravo F. 1089* (SGO). Region IV Coquimbo: Prov. Elqui, valley of Río Turbio between Rivadavia and Guanta, 900 m, 18–19 Jan 1926, *I.M. Johnston 6271* (S); near Guanaqueros, 24 Jul 1973, *O. Zöllner 6827* (L3661655); road from Marquesa to Viñita Baja, 29.954529S, 70.964978W, 340 m, 26 Sep 2017, *F. Luebert, T. Böhnert & F.F. Merklinger 3822* (BONN, ULS); road from Marquesa to Viñita Baja, 29.873778S, 70.860812W, 700 m, 26 Sep 2017, *F. Luebert, T. Böhnert & F.F. Merklinger 3825* (BONN, ULS); road to Andacollo, c. 6 km before Andacollo, 30.201205S, 71.092169W, 900 m, 24 Sep 2017, *F. Luebert, T. Böhnert & F.F. Merklinger 3807* (BONN, ULS). Prov. Limarí, Ovalle, Villaseca near Huamalata, 30.568064S, 71.150966W, 270 m, 24 Sep 2017, *F. Luebert, T. Böhnert & F.F. Merklinger 3799* (BONN, ULS); Fray Jorge, 215 m, 13 Aug 1917, *C. & I. Skottsberg 746* (S); Ovalle, Fray Jorge, 200 m, Nov 1925, *E. Werdermann 892* (U [U1145306], S); Ovalle, Fray Jorge, Oct 1947, *B. Sparre 3061* (S); hotel Termas de Socos, 30.732502S, 71.493507W, 80 m, 23 Sep 2017, *F. Luebert, T. Böhnert & F.F. Merklinger 3777* (BONN, ULS); road from Los Loros to Caleta El Toro, 30.741021S, 71.65348W, 50 m, 23 Sep 2017, *F. Luebert, T. Böhnert & F.F. Merklinger 3790* (BONN, ULS); road from Alcones to Los Loros, 30.78262S, 71.587161W, 330 m, 23 Sep 2017, *F. Luebert, T. Böhnert & F.F. Merklinger 3781* (BONN, ULS). Prov. Choapa, road from Combarbalá to Canela Baja, a few km after Los Pozos, 31.363888S, 71.260395W, 500 m, 22 Sep 2017, *F. Luebert, T. Böhnert & F.F. Merklinger 3775* (BONN, ULS); road from Illapel to Combarbalá, near Illapel, 31.604095S, 71.125953W, 450 m, 22 Sep 2017, *F. Luebert, T. Böhnert & F.F. Merklinger 3772* (BONN, ULS). Region V Valparaíso: Prov. Petorca, La Ligua, 5 km from Petorca on road from Pedequa, 550 m, 27 Nov 1938, *C.R. Worth & J.L. Morrison 16704* (S). Prov. San Felipe de Aconcagua, Cuesta Las Chilcas, 560 m, 19 Jul 2003, *F. Luebert & L. Kritzner 1757* (EIF); Cuesta Las Chilcas, 32.851797S, 70.875068W, 380 m, 20 Sep 2017, *F. Luebert, T. Böhnert & F.F. Merklinger 3741* (BONN, ULS); Cuesta Las Chilcas, 32.851797S, 70.875068W, 380 m, 20 Sep 2017, *F. Luebert, T. Böhnert & F.F. Merklinger 3741A* (BONN, ULS). Prov. Valparaíso, 14 Jan 1947, *E. Wall & B. Sparre 45* (S); rocks near the sea, 17 Nov 1895, *O. Buchtien s.n.* (S). Prov. Quillota, Parque Nacional La Campana, Cerro La Campana, 32°58.777"S, 71°7.670"W, 480 m, 30 Dec 2000, *F. Luebert 1398* (EIF). Prov. San Antonio, Nov 1927, *O. Buchtien 3450* (L3661663). Prov. Melipilla, Curacaví, Dec 1967, (L3661657); La Barriga, Oct 1964, *O. Zöllner 1765* (L3661658).

#### Ophryosporus pinifolius

Taxon classificationPlantaeAsteralesAsteraceae

(Phil.) R.M.King & H.Rob., Phytologia 25: 66. 1972

EC8C263C-7D2B-57A0-9BCA-67C9A8801359

Basionym: Stevia pinifolia Phil., Anales Mus. Nac., Santiago de Chile sec. 2 (bot.) 1891: 37.
Piqueria pinifolia (Phil.) Hieron. ex B.L.Rob., Proc. Amer. Acad. Arts 42: 11 (1906).

##### Type.

Chile. Region I Tarapacá: Usmagama, 15 Mar 1885, *R.A. Philippi s.n.* (lectotype, selected by Plos and Sancho (2013: 339): SGO [SGO044738!]; isolectotype: K [K000486664, photo!]).

*Ophryosporus pinifolius* has an inconspicuous pappus that consists of minute squamellae. Its leaves are extremely variable and range from linear-lanceolate with entire margins to irregularly dentate ones. It is one of two Andean species in the genus known to occur in Chile. Based on herbarium records, its distribution is centered in the northern regions of Tarapacá and Arica y Parinacota where it is widespread (Fig. 1).

##### Specimens examined.

Chile. Region XV Arica y Parinacota: Prov. Arica, Timar, between Timar and Tignamar, 18.717336S, 69.663483W, 2840 m, 29 Mar 2017, *F.F. Merklinger & A. Stoll 2017-51* (BONN, ULS); shortly after Timar, 18.736382S, 69.706777W, 2447 m, 29 Mar 2017, *F.F. Merklinger & A. Stoll 2017-54* (BONN, ULS); shortly after Timar, 18.747992S, 69.699137W, 2307 m, 29 Mar 2017, *F.F. Merklinger & A. Stoll 2017-47* (BONN, ULS); Codpa, between Codpa and Timar, 18.762747S, 69.69828W, 2393 m, 29 Mar 2017, *F.F. Merklinger & A. Stoll 2017-46* (BONN, ULS); quebrada de Vitor NW of Palca, 18.827041S, 69.677724W, 2085 m, 28 Mar 2017, *F.F. Merklinger & A. Stoll s.n.* (BONN, ULS); quebrada Chokaya, from Codpa into Camarones valley toward Pachica, 18.86473S, 69.68034W, 2235 m, 28 Mar 2017, *F.F. Merklinger & A. Stoll 2017-30* (BONN, ULS); quebrada Chokaya, from Codpa into Camarones valley toward Pachica, 18.88289S, 69.664972W, 2373 m, 28 Mar 2017, *F.F. Merklinger & A. Stoll 2017-40* (BONN, ULS); between Esquina and Pachica, 18.927444S, 69.552944W, 2298 m, 28 Mar 2017, *F.F. Merklinger & A. Stoll 2017-43* (BONN, ULS); along road through Illapata, 18.94831S, 69.50272W, 2300 m, 28 Mar 2017, *F.F. Merklinger & A. Stoll 2017-41* (BONN, ULS); road A-135 from Panamericana to Puquios, 18.254661S, 69.828852W, 3100 m, 14 Oct 2017, *F. Luebert, T. Böhnert & F.F. Merklinger 4008* (BONN, ULS). Prov. Parinacota, along road Putre–Arica, 18.21033S, 69.56082W, 3500 m, 1 Apr 2017, *F.F. Merklinger & A. Stoll 2017-65* (BONN, ULS); Paychama [Pachama], 3600 m, 10 Mar 1927, *C. Troll 3244* (B, M); along road Putre–Arica, 18.45314S, 69.76415W, 3102 m, 1 Apr 2017, *F.F. Merklinger & A. Stoll 2017-62* (BONN, ULS); along A-31 near Belen, 18.48545S, 69.52782W, 3500 m, 29 Mar 2017, *F.F. Merklinger & A. Stoll 2017-56* (BONN, ULS); NE of Saxamar, 18.55108S, 69.50015W, 3500 m, 29 Mar 2017, *F.F. Merklinger & A. Stoll 2017-57* (BONN, ULS); Saxamar, 18.56667S, 69.48333W, c. 3000 m, 20 Mar 2015, *A. Moreira & F. Luebert 2456* (BONN); W of Tignamar along road, 18.57918S, 69.52785W, 3300 m, 29 Mar 2017, *F.F. Merklinger & A. Stoll 2017-55* (BONN, ULS); Tignamar, 18°37'S, 69°28'W, 3100 m, 11 Sep 1963, *F. Schlegel 4879* (CONC, F [photo; as *Piqueria floribunda*]); between Timar and Tignamar, shortly before Tignamar, 18.663898S, 69.560963W, 3354 m, 29 Mar 2017, *F.F. Merklinger & A. Stoll 2017-53* (BONN, ULS). Prov. Arica, Palca, 18°50'S, 69°40'W, 2200 m, 30 Oct 1964, *F. Schlegel 5092* (CONC, F [photo, as *Piqueria floribunda*]). Region I Tarapacá: Prov. Tamarugal, quebrada de Soga, 19.514361S, 69.381274W, 2400 m, 16 Mar 2017, *F. Luebert, T. Böhnert & F.F. Merklinger, A. Stoll & D. Quandt 3455* (BONN, ULS); quebrada Aroma, 19.514361S, 69.381274W, 2300 m, 16 Mar 2017, *F.F. Merklinger 2017-6* (BONN, ULS); above Jaiña, 19.551255S, 69.242848W, 2750 m, 21 Mar 2017, *F. Luebert & T. Böhnert 3631* (BONN, ULS); at entrance of Andean village Chusmiza, 19.67830S, 69.17930W, 3393 m, 2 Oct 2019, *F.F. Merklinger, F. Luebert & J. Ruhm 2019-21* (BONN, EIF, K); Chusmiza, above town, 19.683025S, 69.183376W, 3400 m, 21 Mar 2017, *F. Luebert & T. Böhnert 3642* (BONN, ULS); 2 km above the village of Chusmiza in quebrada de Chusmiza at km 76 alongside the new road to Colchane, 19°40'53.7"S, 69°11'11.0"W, 3406 m, 18 Feb 2003, *M.F. Gardner & S.G. Knees 6512* (SGO150393); road to Usmagama, turnoff ruta CH-15, 19.730253S, 69.218684W, 2956 m, 26 Mar 2017, *F.F. Merklinger & A. Stoll 2017-26* (BONN, ULS); road to Usmagama, turnoff ruta CH-15, 19.730253S, 69.218684W, 2956 m, 26 Mar 2017, *F.F. Merklinger & A. Stoll 2017-19* (BONN, ULS); cuesta Usmagama, km 3.9, 19.730154S, 69.217046W, 3050 m, 28 Oct 2016, *F. Luebert & T. Böhnert 3452* (BONN, ULS); Usmagama, road from Usmagama to Limacsina, 19.78771S, 69.207368W, 2434 m, 26 Mar 2017, *F.F. Merklinger & A. Stoll 2017-22* (BONN, ULS); quebrada de Parca, 19.985261S, 69.098117W, 3261 m, 22 Mar 2017, *F.F. Merklinger & A. Stoll 2017-12* (BONN, ULS).

#### Ophryosporus triangularis

Taxon classificationPlantaeAsteralesAsteraceae

Meyen, Reise Erde 1: 402. 1834

80E4D9E9-6B0A-56F1-A3B3-9D84A76A3E23

Eupatorium decipiens Hook. & Arn. in Hook. Compan. Bot. Mag. 1: 240. 1835. Type: Chile. Region IV Coquimbo: *H. Cuming 907* (lectotype, designated here, K [K000486661, photo!]; isolectotype: E [E00249908, photo!]); remaining syntypes: Chile. Region IV Coquimbo: *Macrae s.n.* E [E00249907, photo!, mounted together with lectotype].
Eupatorium foliolosum DC., Prodr. 5: 174. 1836.
Ophryosporus foliolosus (DC). Reiche, Anales Univ. Chile 109: 9. 1901. Type: Chile. Region IV Coquimbo: *Macrae s.n.* (lectotype, designated here: G-DC [G00130591, photo!]; remaining syntypes: *Gaudichaud 100* (G-DC [G00130590, photo!]; P [P02673049 & P02673151, photo!]; K [K486660 & K486663, photo!]).
Kuhnia multiramea Turcz. Bull. Soc. Imp. Naturalistes Moscou 24: 168. 1851. Type: Chile. Region IV Coquimbo: *T.C. Bridges 1412* (holotype: KW [144506, photo!]; isotypes: KW [144505, photo!]; E [E00249909, photo!]; P [P02673149, photo!]).
Eupatorium volckmannii Phil. Anales Univ. Chile 18: 51. 1861. Type: Chile. Region III Atacama, Vallenar: *Volckmann s.n.* 1860 (holotype: SGO [SGO065417!]; isotype: GH [GH00014290, photo!]).

##### Type.

Chile. Region III Atacama: Copiapó, *F.J.F. Meyen s.n.* (holotype: B [probably destroyed]; F neg. 14718!).

##### Notes.

This species is widespread and more or less continuously distributed along the coast of northern Chile. The southernmost localities where it was observed during our study were at the southern edge of the Río Limarí, where its range overlaps with that of *Ophryosporus paradoxus*. The northern end of its distribution appears to be the Río Loa. North of Paposo, at Aguada del Panul, it grows sympatrically with *O. johnstonii*, to which it bears close morphological resemblance. However, *O. triangularis* is identified by its slightly larger leaves, which are grouped in alternating fascicles, are shortly petiolate, triangular with a cuneate base and an acute apex, and reaching about 3–5 × 5–15 mm as opposed to much smaller leaves in *O. johnstonii*, which reach only c. 1–3 × 3–5 mm. Leaf-size is, however, extremely variable, and plants that grow in more humid conditions often possess larger leaves. The leaf margins of *O. triangularis* are regularly lobed to dentate and revolute. The inflorescences are spike-like, and the capitula are pedunculated. The spike-like inflorescences appear somewhat denser than in *O. johnstonii*, and, in this latter species, the capitula are sessile. Its florets have a white corolla, sometimes with a violet taint. The pappus is formed by whitish-brown setae, c. 2.5–3.5 mm long. In the area around Cobija, both, *O. triangularis* and *O. anomalus* have been collected in the past.

##### Specimens examined.

Chile. Region II Antofagasta: Prov. Tocopilla, quebrada 2–3 km N of Tocopilla above old Caleta Duendes, 150–200 m, 18 Oct 1988, *M.O. Dillon & D. Dillon 5718* (BONN); Tercera quebrada Tocopilla, 22.0558S, 70.17662W, 300 m, 18 Oct 2016, *F. Luebert, A. Stoll & T. Böhnert 3413* (BONN, ULS); Quebrada La Higuera, S Tocopilla, 22°18'7"S, 70°12'58"W, 30 Sep 2005, *F. Luebert, N. Garcia & N. Schulz 2569/963* (EIF); Quebrada above Caleta Buena, S Mantos de la Luna, 22.43075S, 70.22186W, 640 m, 10 Oct 2017, *F. Luebert, T. Böhnert & F.F. Merklinger 3989* (BONN, ULS). Prov. Antofagasta, near Cobija, 19 Dec 1971, *O. Zöllner 4593* (L153863); Juan López, 23.51205S, 70.53365W, 150 m, 19 Jul 2003, *M. Antonissen 7* (EIF); Quebrada La Chimba, 17 Dec 1987, *M.O. Dillon & J.T.S. Teillier 5321* (BONN); Quebrada La Chimba, 23°33'S, 70°22'W, 380–480 m, 11 Nov 1988, *M.O. Dillon & D. Dillon 5881* (BONN); Quebrada La Chimba, 23.53567S, 70.35887W, 460 m, 17 Oct 2016, *F. Luebert, A. Stoll & T. Böhnert 3408* (BONN, ULS); Quebrada Cardón, 24.741717S, 70.542687W, 210 m, 5 Oct 2017, *F. Luebert, T. Böhnert & F.F. Merklinger 3949* (BONN, ULS); Aguada Cardón, 24.74173S, 70.54385W, 210 m, 15 Oct 2016, *F. Luebert, A. Stoll & T. Böhnert 3389* (BONN, ULS); Quebrada Panul, 24.773468S, 70.533915W, 180 m, 4 Oct 2017, *F. Luebert, T. Böhnert & F.F. Merklinger 3939* (BONN, ULS); Quebrada Panul, 24.777263S, 70.531618W, 190 m, 5 Oct 2017, *F. Luebert, T. Böhnert & F.F. Merklinger 3944* (BONN, ULS); Quebrada Panul, 24.777263S, 70.531618W, 190 m, 5 Oct 2017, *F. Luebert, T. Böhnert & F.F. Merklinger 3944A* (BONN, ULS); c. 7 km N of Paposo, 24°57'S, 70°29'W, 40 m, 14 Dec 1987, *M.O. Dillon & J.T.S. Teillier 5262* (BONN); El Rincón, al N de Paposo, 17 Sep 1941, *C. Muñoz P. & G.T. Johnson 2902* (SGO118350); Paposo, base Cerro Carneros, 24°56'24"S, 70°28'44.6"W, 160 m, 23 Oct 2009, *A. Moreira & F. Luebert 1200* (SGO158780); Quebrada Portezuelo, 25.0124S, 70.446467W, 550 m, 13 Oct 2016, *F. Luebert, A. Stoll & T. Böhnert 3357* (BONN, ULS); Quebrada Matancilla, c. 5 km S of Punta Grande, 25°07'S, 70°27'W, 170–350 m, 27 Oct 1988, *M.O. Dillon, D. Dillon, V. Asencio & M. Villarroel O. 5750* (BONN); Cachinalcito, 25°10', 28 Nov 1925, *I.M. Johnston 5173* (S); c. 20 km N of Taltal, quebrada Anchuña, 25.23543S, 70.42594W, 183 m, 31 Aug 2018, *F.F. Merklinger, A. Kozok & D. Quandt 2018-63* (BONN, ULS); c. 20 km N of Taltal, quebrada Anchuña, 25.23543S, 70.42594W, 183 m, 31 Aug 2018, *F.F. Merklinger, A. Kozok & D. Quandt 2018-64* (BONN, ULS); c. 20 km N of Taltal, quebrada Anchuña, 25.23543S, 70.42594W, 183 m, 31 Aug 2018, *F.F. Merklinger, A. Kozok & D. Quandt 2018-65* (BONN, ULS); c. 20 km N of Taltal, quebrada Anchuña, 25.23543S, 70.42594W, 183 m, 31 Aug 2018, *F.F. Merklinger, A. Kozok & D. Quandt 2018-66* (BONN, ULS); Quebrada El Médano, 300 m, 8 Oct 1941, *E. Pisano V. & R. Bravo F. 398* (SGO); Quebrada San Ramón, 25.38578S, 70.43658W, 120 m, 11 Oct 2016, *F. Luebert, A. Stoll & T. Böhnert 3346* (BONN, ULS); Quebrada San Ramón, 17 Sep 1968, *O. Zöllner 2851* (U [U3661650]); Cerro Perales, c. 5 km E of Taltal, 25°25'S, 70°25'W, 550 m, 21 Nov 1988, *M.O. Dillon & D. Dillon 6002* (BONN); Hills SE of Taltal, 25°29'S, 25 Nov 1925, *I.M. Johnston 5080* (S); Posado Hidalgo, 25°45'S, 70°35'W, 13 Dec 1925, *I.M. Johnston 5661* (S); along road Panamericana toward Caleta Esmeralda, 25.895921S, 70.581052W, 500 m, 9 Oct 2016, *F. Luebert, A. Stoll & T. Böhnert 3308* (BONN, ULS). Region III Atacama: Prov. Chañaral, 21 km W of Panamericana on northern route to Pan de Azucar NP, 26°08'S, 70°37'W, 85 m, 30 Sep 1988, *M.O. Dillon, D. Dillon & V. Pobleto 5609* (BONN); Falda Verde, hills N of Chañaral, 26.296721S, 70.631252W, 75–600 m, 1 Oct 2017, *F. Luebert, T. Böhnert & F.F. Merklinger 3905* (BONN); Hills back of El Barquito, 26°23'S, 28–29 Oct 1925, *I.M. Johnston 4809* (S). Prov. Copiapó, Sector quebrada El León, 26.976625S, 70.773903W, 70 m, 8 Oct 2016, *F. Luebert, A. Stoll & T. Böhnert 3290* (BONN, ULS); Caldera on small point just north of town, 27°3'S, 22 Nov 1925, *I.M. Johnston 5067* (S); Copiapó, 400 m, 13 Jul 1938, *Ch.H. Andreas 885* (U [U1145297]); Tierra Amarilla, 700 m, Oct 1924, *E. Werdermann 456* (U [U1145305]); Estancia Castilla, road from Totoral to Panamericana, 27.919994S, 70.84531W, 240 m, 28 Sep 2017, *F. Luebert, T. Böhnert & F.F. Merklinger 3869* (BONN, ULS). Prov. Huasco, Road Carrizal Bajo to Canto de Agua, c. 2 km from Carrizal Bajo, 28.112004S, 71.116139W, 45 m, 7 Oct 2016, *F. Luebert, A. Stoll & T. Böhnert 3279* (BONN, ULS); Quebrada Baratillo, 28°21'57"S, 71°7'21"W, 150 m, 14 Sep 2003, *F. Luebert & L. Kritzner 1805* (EIF); Quebrada Baratillo, 28.363325S, 71.096321W, 110 m, 7 Oct 2016, *F. Luebert, A. Stoll & T. Böhnert 3269* (BONN, ULS); Hills E of Tres Playitas, 28.400532S, 71.16755W, 160 m, 27 Sep 2017, *F. Luebert, T. Böhnert & F.F. Merklinger 3854* (BONN, ULS). Region IV Coquimbo: Prov. Elqui, Guayacán, s.a., *unknown s.n.* (S); Puente Juan Soldado, 29.656542S, 71.301174W, 200 m, 27 Sep 2017, *F. Luebert, T. Böhnert & F.F. Merklinger 3831* (BONN, ULS); La Serena, 16 Sep 1947, *B. Sparre 2595* (S); Coquimbo, Jul–Aug 1958, *W.H. Harvey s.n.* (S); Coquimbo, 100 m, Nov 1923, *E. Werdermann 124* (U [U1145298]); Coquimbo, rocks behind the Fort, 29.93372S, 71.33691W, 25 m, 24 Sep 2017, *F. Luebert, T. Böhnert & F.F. Merklinger 3809* (BONN, ULS); Coquimbo, rocks behind the Fort, 29.93372S, 71.33691W, 25 m, 24 Sep 2017, *F. Luebert, T. Böhnert & F.F. Merklinger 3809A* (BONN, ULS); Herradura, 9 Aug 1917, *C. & I. Skottsberg 704* (S). Prov. Limarí, Ovalle, Río Límari, 11 Oct 1947, *B. Sparre 2994* (S); N of Caleta El Toro, 30.737239S, 71.699907W, 25 m, 23 Sep 2017, *F. Luebert, T. Böhnert & F.F. Merklinger 3794* (BONN, ULS); along road from Los Loros to Caleta El Toro, 30.741021S, 71.65348W, 50 m, 23 Sep 2017, *F. Luebert, T. Böhnert & F.F. Merklinger 3786* (BONN, ULS).

### Species excluded from the Chilean flora

#### Ophryosporus floribundus

Taxon classificationPlantaeAsteralesAsteraceae

(DC.) R.M.King & H.Rob., Phytologia 25: 66. 1972

E63E026F-860E-5C3A-AFE1-F4E9F7CAB5B9

Basionym: Piqueria floribunda DC., Prodr. 5: 105. 1836.

##### Type.

Peru. “Perou cordilliere, 1834, *T.P.X. Haenke s.n.* (holotype: G-DC [G00130596, photo!]; isotype: P [P00742191, photo!]).

This taxon is cited for Chile in Muñoz-Schick et al. (2001), Pinto and Luebert (2009), Zuloaga et al. (2008) and Rodríguez et al. (2018) based on four specimens. Three of them were collected by R. Pinto in the late 1990s at three coastal localities in northern Chile, Alto Chipana, Punta Lobos and Punta Gruesa (*Pinto, s.n.*, SGO 142948, SGO 142949 and SGO 142950). Recent field work at the coastal localities has not resulted in any collections that match the type of *O. floribundus.* Rather we found plants that we identified as *O. anomalus*, another species that has been reported for this area (Johnston 1932) and has been only sporadically collected since. The type of *O. floribundus* has opposite, solitary leaves and long internodes of about 3–4 cm in length. The type of *O. anomalus* has crowded leaves that are borne in fascicles and with very short internodes. The leaves of the Chilean coastal specimens assigned to *O. floribundus* vary in size and shape, some corresponding well to the type of *O. anomalus* being narrowly oblanceolate and with entire margins and an obtuse apex, others becoming more broadly lanceolate to triangular with dentate margins and an acute apex thus remotely resembling *O. floribundus* but actually more similar to those of *O. triangularis*. On younger shoots the leaves appear more or less opposite but generally they are borne in fascicles. The cypselae of the plants on both sheets of the type specimen of *O. anomalus* (*Cuming 953*, K) bear quite visible, slightly spreading squamellate pappus setae, while no pappus is recorded for *O. floribundus.* We therefore conclude that *O. floribundus* is restricted to Peru from the area of Lima, while the coastal plants in northern Chile should be referred to *O. anomalus*.

##### Specimens examined.

Peru. Dept. Amazonas: Purruchuca [sic], s.a., *Mathews, A. 1015* (G). Dept. Lima: Prov. Canta, road from Canta to Lima below San José turnoff towards Lima, 11.49383S, 76.65187W, 2322 m, 1 Mar 2018, *M. Weigend & K.A. Peña Ramos 9719* (BONN); road from Canta to Lima, road down from Canta to turn off San José, 11.49383S, 76.65187W, 2322 m, 1 Mar 2018, *M. Weigend & K.A. Peña Ramos 9722* (BONN). Prov. Huarochiri, between Matucana and Tambo, 26 Dec 1901, *Weberbauer, A. 115* (G); Matucana, c. 2400 m, 25 May 1940, *E. Asplund 11072* (S); Matucana, c. 2600 m, 25 May 1940, *E. Asplund 11079* (S).

#### Ophryosporus hoppii

Taxon classificationPlantaeAsteralesAsteraceae

(B.L.Rob.) R.M.King & H.Rob., Phytologia 23: 399. 1972

5A65173D-5690-5FB9-A921-A240638B0A25

Basionym: Trychinolepis hoppii B.L.Rob., Contr. Gray Herb. 80: 6. 1928.

##### Type.

Peru. Dept. Arequipa: Jul 1925, *W. Hopp 28* (holotype: B [probably destroyed], F neg. 14723!; lectotype, designated here: GH [GH00013302]). Epitype (designated here): Peru. Dept. Arequipa. Lomas of Mollendo, c. 4 km N of Islay, 230 m, 20 Nov 1983, *M.O. Dillon & D. Dillon 3926* (USM [74666]; isoepitypes: BONN!, F!, US [3026292, photo!]).

This taxon was originally described as a new genus and species, *Trychinolepis hoppii* (Robinson 1928: 6), because of its irregularly lobed, squamellate pappus, which resembled that of the West Indian genus *Phania*, even though a habitual resemblance to the genus *Ophryosporus* was stated by the author (Robinson 1928). Subsequent analyses led to this monotypic genus to be allocated to *Ophryosporus*, because the pappus remained the only difference with other species of *Ophryosporus* (King and Robinson 1972a). Specimens assigned to *O. hoppii* in Peru have a squamellate pappus, where the setae are fused at the base and are rather conspicuous. Cypselae with this type of pappus are visible on the type specimen and can even be seen on online images. However, due to the loss of the holotype in B and the very fragmentary remnants of the isotype at GH, we have decided to epitypify the specimen collected by M.O. Dillon and D. Dillon in 1983, as it corresponds to the protologue of *T. hoppii* and has been collected in the same region. This specimen has all necessary characters suitable for identification and is a good reference for potential future work.

Specimens which we could positively identify as *Ophryosporus hoppii* were all collected in Peru. Records of *O. hoppii* for Chile appear to be miss-identifications and belong to *O. pinifolius*, e.g. *Schlegel 4879* & *5092* (CONC, F). The pappus of *O. pinifolius* on the contrary, although squamellate, are much more inconspicuous (Fig. 2). Our own observations and extensive sampling in northern Chile failed to positively identify a plant that clearly fits into the species concept of *O. hoppii*. For example, a specimen from Chusmiza, N of Iquique (*O. Zöllner 2997*, U) is cited twice in Plos (2012), once for *O. hoppii* and a second time for *O. heptanthus*. The locality at Chusmiza was visited by the authors, and two species, *O. pinifolius* and *O. heptanthus*, were confirmed. These two taxa grow in local sympatry here, yet are distinguished from one another by their pappus, which in *O. pinifolius* consists of minute squamellae and which in *O. heptanthus* is formed by setae, up to 4 mm long. It is possible that the two taxa were collected by Zöllner as one species, only for later herbarium identification to recognize the two different taxa in the two herbarium vouchers. A further herbarium record from Alto Patache in Chile (*H.Larraín 98200*, CONC) could not be found in CONC.

##### Specimens examined.

Peru. Dept. Arequipa: Prov. Islay, Quebrada Guerreros, 456 m, 6 Apr 1998, *FLSP 2411* (HUSA, US);Ocoña, 5 Feb 1969, *J. Soukup 6426/6216* (US, USM). Prov. Castilla, Chuquibamba towards Aplao 15°51'52.3"S, 72°36'56.4"W, 2231 m, 24 Mar 2019, *M. Weigend 9862/19-52* (BONN, USM).

## Supplementary Material

XML Treatment for Ophryosporus anomalus

XML Treatment for Ophryosporus heptanthus

XML Treatment for Ophryosporus johnstonii

XML Treatment for Ophryosporus paradoxus

XML Treatment for Ophryosporus pinifolius

XML Treatment for Ophryosporus triangularis

XML Treatment for Ophryosporus floribundus

XML Treatment for Ophryosporus hoppii

## Supplementary materials

Supplementary material 1 Table S1 Data type excel table in xlsx format Explanation note Complete georeferenced specimen data of all vouchers considered in this study. https://binary.pensoft.net/file/452773This dataset is made available under the Open Database License (http://opendatacommons.org/licenses/odbl/1.0/). The Open Database License (ODbL) is a license agreement intended to allow users to freely share, modify, and use this Dataset while maintaining this same freedom for others, provided that the original source and author(s) are credited.Felix F Merklinger, Federico Luebert

## References

[B1] Brako L Zarucchi JL (1993) Catalogue of the flowering plants and gymnosperms of Peru. Monographs in Systematic Botany from the Missouri Botanical Garden 45: 1–1286.

[B2] Hind DJN Robinson H (2007) Tribe Eupatorieae. In: Kubitzki K Kadereit JW Jeffrey C (Eds) The Families and Genera of Vascular Plants.Volume VIII, Flowering Plants, Eudicots: Asterales. Springer, Berlin, New York, 510–574.

[B3] Jaffuel PF (1936) Excursiones botánicas a los alrededores de Tocopilla. Revista Chilena de Historia Natural 40: 265–274.

[B4] Johnston IM (1929) Papers on the flora of Northern Chile. Contributions from the Gray Herbarium of Harvard University 85: 1–180.

[B5] Johnston IM (1932) New records for the flora of the Nitrate Coast. Revista Chilena de Historia Natural 36: 4–8.

[B6] King RM Robinson H (1972a) Studies in the Eupatorieae (Asteraceae). LXXIII. The genus *Ophryosporus*. Phytologia 23: 397–400. 10.5962/bhl.part.19873

[B7] King RM Robinson H (1972b) Studies in the Eupatorieae (Asteraceae). CXI. Additions to the genus *Ophryosporus*. Phytologia 25: 65–67.

[B8] Kunth KS (1818) Nova genera et species plantarum, vol. 4 [Folio ed.]. Libraria Graeco-Latino-Germanica, Paris, 246 pp.

[B9] Luebert F García N Schulz N (2007) Observaciones sobre la flora y vegetación de los alrededores de Tocopilla (22°S, Chile). Boletín del Museo Nacional de Historia Natural 56: 27–52.

[B10] Muñoz-Schick M Pinto R Mesa A Moreira-Muñoz A (2001) “Oasis de neblina” en los cerros costeros del sur de Iquique, región de Tarapacá, Chile, durante el evento El Niño 1997–1998. Revista Chilena de Historia Natural 74(2): 389–405. 10.4067/S0716-078X2001000200014

[B11] Pinto R Luebert F (2009) Datos sobre la flora vascular del desierto costero de Arica y Tarapacá, Chile, y sus relaciones fitogeograficas con el sur de Peru. Gayana. Botánica 66(1): 28–49. 10.4067/S0717-66432009000100004

[B12] Plos A (2012) Revisión sistemática, análisis cladístico y biogeográfico del género *Ophryosporus* Meyen (Asteraceae, Eupatorieae, Critoniinae). Unpublished thesis, Universidad Nacional de La Plata.

[B13] Plos A Sancho G (2013) Lectotipificaciones en *Ophryosporus* (Asteraceae, Eupatorieae, Critoniinae). Boletín de la Sociedad Argentina de Botánica 48(2): 335–340. 10.31055/1851.2372.v48.n2.6267

[B14] Robinson BL (1906) Studies in the Eupatorieae. II. Revision of the genus *Ophryosporus*. Proceedings of the American Academy of Arts and Sciences 42: 17–27. 10.2307/20022173

[B15] Robinson BL (1928) Records preliminary to a general treatment of the Eupatorieae – VII. Contributions from the Gray Herbarium of Harvard University 80: 6–7.

[B16] Rodriguez R Marticorena C Alarcón D Baeza C Cavieres L Finot VL Fuentes N Kiessling A Mihoc M Pauchard A Ruiz E Sanchez P Marticorena A (2018) Catálogo de las plantas vasculares de Chile. Gayana. Botánica 75(1): 1–430. 10.4067/S0717-66432018000100001

[B17] Zuloaga FO Morrone O Belgrano MJ (2008) Catálogo de las plantas vasculares del Cono Sur (Argentina, Sur de Brasil, Chile, Paraguay y Uruguay). Vol. 2, Acanthaceae-Fabaceae. Monographs in Systematic Botany from the Missouri Botanical Garden 107: 1–3348. [1417–1418]

